# *In vitro *atovaquone/proguanil susceptibility and characterization of the *cytochrome b *gene of *Plasmodium falciparum *from different endemic regions of Thailand

**DOI:** 10.1186/1475-2875-7-23

**Published:** 2008-01-28

**Authors:** Rommanee Khositnithikul, Peerapan Tan-ariya, Mathirut Mungthin

**Affiliations:** 1 Department of Microbiology, Faculty of Science, Mahidol University, Rama VI Rd, Bangkok 10400, Thailand; 2 Department of Parasitology, Phramongkutklao College of Medicine, Ratchawithi Rd, Bangkok 10400, Thailand

## Abstract

**Background:**

The emergence of *Plasmodium falciparum *resistant to most currently used antimalarial drugs is the major problem in malaria control along the Thai-Myanmar and Thai-Cambodia borders. Although artemisinin-based combination therapy has been recommended for the treatment of multidrug-resistant falciparum malaria, these combinations are not available for some people, such as travelers from North America. A fixed-dose combination of atovaquone and proguanil (Malarone) has been proved to be effective for the treatment and prophylaxis of malaria which is already approved by countries in North America and Europe. Determination of the phenotypes and genotypes related to atovaquone/proguanil response in Thai isolates of *P. falciparum *will be useful for rationale drug use. The main purpose of this study was to explore the *in vitro *atovaquone/proguanil susceptibility of recently adapted Thai isolates of *P. falciparum*. Genotypic characterization of the *cytb *gene of these isolates was also determined since it has been reported that point mutations, particularly codon 268 in the cytochrome b gene (*cytb*) have been linked to atovaquone/proguanil treatment failure.

**Methods:**

Eighty three *P. falciparum *isolates collected during 1998 to 2005 from four different multidrug resistance areas of Thailand were determined for the *in vitro *atovaquone/proguanil susceptibilities using radioisotopic assay. Mutations in the *cytb *gene were determined by PCR-RFLP and sequence analysis.

**Results:**

The mean atovaquone and proguanil IC_50 _was 3.4 nM and 36.5 μM, respectively. All 83 Thai isolates were atovaquone sensitive. None of the 83 isolates contained the mutations at codon 268 of the *cytb *gene. DNA sequencing of the *cytb *gene of 20 parasite isolates showed no other mutations.

**Conclusion:**

In agreement with a recent efficacy study of atovaquone/proguanil, the present information indicates that atovaquone/proguanil can be one of the drugs of choice for the treatment and prophylaxis of multidrug-resistant falciparum malaria in Thailand.

## Background

Multidrug-resistant falciparum malaria is a major health problem along Thai-Myanmar and Thai-Cambodia borders. WHO has recommended artemisinin-based combination therapy as the first-line treatment for uncomplicated falciparum malaria in these multidrug resistance areas. In Thailand, a combination of artesunate and mefloquine has been used for more than 10 years. Recently, reduced efficacy of this combination has been reported from Thai-Cambodia border [1]. Effective chemoprophylaxis for malaria in these multidrug resistance areas is also needed especially for non-immune travelers. Thus the alternative drugs or drug combinations should be considered. Malarone^® ^is a fixed-dose combination of atovaquone and proguanil. It is highly effective for the treatment and prophylaxis of multidrug-resistant falciparum malaria [2,3]. Atovaquone exerts its action via inhibiting plasmodial mitochondria electron transport at the level of the cytochrome bc_1 _complex and collapsing mitochondrial membrane potential [4,5]. In addition, it inhibits dihydroorotate dehydrogenase (DHOD) enzyme which catalyses the reaction from dihydroorotate to orotate [6]. Therefore, atovaquone can inhibit both nucleic acid and ATP synthesis. Proguanil was added for the synergistic effect which is probably due to enhancing atovaquone to collapse mitochondrial membrane potential [7].

A few cases of atovaquone-proguanil (AP) treatment failure have been reported, mainly from Africa [8-16]. Treatment failure of AP in these patients was due to atovaquone resistance and has been linked to point mutations in the target gene of atovaquone, *Plasmodium falciparum *mitochondrial *cytb *gene, i.e. Tyr268Ser, Tyr268Asn and Tyr268Cys. Codon 268 of the *cytb *gene is a region encoding the putative atovaquone-binding domain [17]. Tyrosine (Y) at position 268 is conserved bulky hydrophobic contact of the drug in the Qo II region of the ubiquinol oxidation site. Substitution of serine (S), a hydrophilic amino acid, limits hydrophobic contact with atovaquone. This reason explains the marked decrease in atovaquone susceptibility in mutated malaria parasites [8,17]. Substitution of the less bulky asparagine (N) at position 268 not only reduced the volume of the binding pocket but it also decreases the affinity and binding of atovaquone [8]. Thus, these mutations are considered as the main markers for the surveillance of AP resistance. To date, AP has not been routinely used for the treatment and prophylaxis of falciparum malaria in Thailand. However, recent study showed a high efficacy of AP for the treatment of multidrug-resistant falciparum malaria on Thailand-Myanmar border [18]. This combination may be considered as the alternative drugs for the treatment and prophylaxis of malaria especially for western non-immune travelers. However, surveillance of AP resistance at the phenotypic and genotypic levels is necessary before this combination is recommended. Since recent report showed that AP-resistant genotype of *P. falciparum *can be detected in the non-exposed area. Happi *et al *found Tyr268Asn mutation in *P. falciparum *from Nigeria where AP has not been used; his finding suggests that the mutations in the *cytb *gene might naturally occur [19]. This study aimed to determine the *in vitro *atovaquone and proguanil sensitivity of adapted Thai isolates of *P. falciparum *from four different endemic areas. Genotypic characterization of the *cytb *gene of these isolates was also performed.

## Methods

### Malaria parasites

Eighty three isolates of *P. falciparum *were collected from four endemic areas along Thai-Myanmar border (Tak, Kanchanaburi and Ranong) and Thai-Cambodia border (Chantaburi) during 1998 to 2005. These isolates were adapted and cryopreserved in liquid nitrogen before used. Parasites were cultivated continuously *in vitro *by a modification of Trager and Jensen method [20].

### *In vitro *drug sensitivity assay

*In vitro *atovaquone and proguanil susceptibility of these Thai isolates of *P. falciparum *was determined using modified radioisotopic method of Desjardins *et al *[21]. Both atovaquone and proguanil were kindly provided by Stephen A Ward (Liverpool School of Tropical Medicine, UK). Atovaquone was dissolved in dimethylsulfoxide (DMSO), whereas proguanil was dissolved in 50% ethanol. The IC_50 _was eventually evaluated using the GRAFIT^® ^programme (Erithacus Software Ltd., UK). The IC_50 _value of each isolate was the mean of at least three independent experiments. Comparisons of the IC_50 _values of parasites collected in different years and from different areas were analyzed using One-way ANOVA. The level of significance was set at *p *< 0.05.

### PCR-RFLP for detection of mutations in the *cytb *gene

*P. falciparum *DNA was isolated using chelex-resin as described by Wooden *et al *[22]. PCR-RFLP was performed to determine the three known polymorphisms at codon 268 of the *cytb *gene using primers CYTB3/CYTB5, CYTB2/CYTB6 and CYTB2/CYTB7 as previously described [9]. For RFLP analysis, the PCR products of each pair of primers were digested with *Nsi*I, *AlwN*I and *Ssp*I, respectively (New England Biolabs, UK). *Nsi*I cut the wild type and asparagine mutation. *AlwN*I cut the serine mutation while *Ssp*I cut wild type and serine mutation but not the asparagine mutation. The digested PCR products were subsequently analyzed by agarose gel electrophoresis.

### DNA sequencing of the *cytb *gene

Since the point mutations in the *cytb *gene at the codons other than 268, i.e. 133 and 284 were previously reported to be involved with atovaquone sensitivity, DNA sequencing of the *cytb *gene was also performed. Twenty isolates were randomly selected from 83 isolates collecting from different years and endemic areas. These 20 isolates also represented parasites with the highest and lowest atovaquone IC_50 _values. The primers pair of CYTB1 and CYTB2 was used for PCR amplification. The PCR products were sequenced by Macrogen Inc, Korea.

## Results and Discussion

### *In vitro *atovaquone and proguanil susceptibility

**Figure 1:**
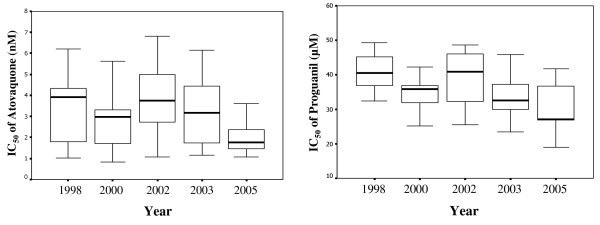
Box plots of atovaquone and proguanil IC_50 _of parasite isolates collected in different years.

**Figure 2:**
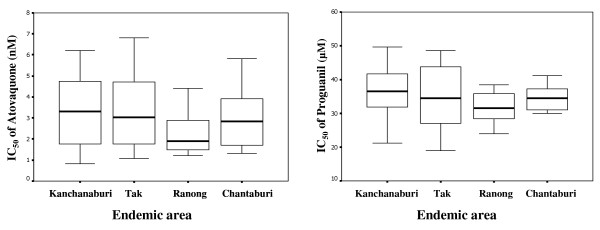
Box plots of atovaquone and proguanil IC_50 _of parasite isolates collected from different endemic areas.

The mean atovaquone IC_50 _values of *P. falciparum *isolates in this study was 3.4 ± 1.6 (0.83–6.81 nM). While, the mean proguanil IC_50 _values was 36.5 ± 7.0 (21.2–49.6). The atovaquone and proguanil IC_50 _values of *P. falciparum *isolates obtained in various years and from various endemic areas were shown in Figure 1 and 2, respectively. The mean ± SD of atovaquone IC_50 _values in 1998, 2000, 2002, 2003 and 2005 were 3.4 ± 1.6, 2.8 ± 1.6, 3.8 ± 1.5, 3.2 ± 1.6 and 2.0 ± 0.8 nM, respectively. The mean ± SD of proguanil IC_50 _values in 1998, 2000, 2002, 2003 and 2005 were 39.9 ± 7.8, 34.8 ± 5.1, 39.2 ± 7.4, 33.8 ± 5.9 and 29.8 ± 7.9 μM, respectively. The mean ± SD of atovaquone IC_50 _value from Tak, Kanchanaburi, Ranong and Chantaburi were 3.4 ± 1.6, 3.3 ± 1.6, 2.9 ± 1.5 and 2.6 ± 1.6 nM, respectively. The mean ± SD of proguanil IC_50 _value from Tak, Kanchanaburi, Ranong and Chantaburi were 36.7 ± 7.28, 35.0 ± 9.3, 34.9 ± 4.3 and 31.7 ± 4.7 μM, respectively. The cut-off point used for atovaquone sensitive and atovaquone resistance was the IC_50 _of <30 nM and >1900 nM, respectively [15]. Based on these criteria, all 83 Thai isolates were sensitive to atovaquone. Although the mean atovaquone IC_50 _value of these Thai isolates was apparently higher than African isolates [15,23], this IC_50 _value (3.2 nM) was still classified in the range of atovaquone sensitive. Futhermore Gay *et al *determined the correlation of atovaquone and other antimalarial drugs in parasites isolated from the Philippines and found the significant correlations between of the IC_50 _of atovaquone and chloroquine, quinine, mefloquine, artemisinin and its derivatives [23]. The sensitivity of 83 isolates to other drugs was also determined and showed that there was no correlation among atovaquone and chloroquine, quinine, mefloquine and dihydroartemisinin IC_50_. The absence of the correlation of atovaquone and most available drugs in these isolates is a good evidence for the possibility of using AP as the alternative antimalarial drug in Thailand.

In this study, both atovaquone and proguanil sensitivity of these isolates were gradually decreased over the seven years of collection period. There were significantly differences between the proguanil IC_50 _values in the years 1998 and 2005 (*p *= 0.021) and in the year 2002 and 2005 (*p *= 0.020). There was no difference of the atovaquone and proguanil IC_50 _values among the isolates adapted from different endemic areas.

### PCR-RFLP and sequencing

Regarding to atovaquone, it has been reported that the mutations in the *cytb *gene especially at the codon 268 is responsible for atovaquone resistance [8-16]. In this study, all isolates were identified as wild-type genotype of the *cytb *gene at the codon 268. Sequence analysis also confirmed that all selected 20 isolates had a wild-type *cytb *gene at the codon 268. Recent studies showed a low prevalence of polymorphisms at the codon 268 [13,24]. These point mutations at the codon 268 seems to be sufficient, but not necessary, for AP treatment failure since the point mutations at this position were not identified in every treatment failure cases [13]. Other mutations i.e. M133I and V284K have been also linked to atovaquone resistance. From sequence analysis, there was no M133I and V284K mutation in all 20 isolates. The results of DNA sequencing were accorded well with the results of *in vitro *drug sensitivity assay and suggested that there was no atovaquone-resistant phenotype and genotype in these Thai isolates of *P. falciparum*. Similar results were previously reported in a study of parasite isolates from Thai-Myanmar border by Naoshima-Ishibashi *et al*; all samples showed no mutations at the codon 268 of the *cytb *gene [25].

A recent efficacy study also showed that AP remained highly efficacious for the treatment of multidrug-resistant falciparum malaria in Thailand [18]. From both *in vitro *and *in vivo *information, AP can be considered as the drug of choice for the treatment and prophylaxis of falciparum malaria in Thailand.

## Conclusion

The evidence from our study strongly supports the possibility of AP as an alternative antimalarial drug for the treatment and prophylaxis of multidrug-resistant falciparum malaria in Thailand.

## Authors' contributions

PT and MM contributed to the conception and design of the study. RK performed *in vitro *susceptibility test and genotyping. RK, PT and MM analysed the data and wrote the manuscript. All authors read and approved the final version that was submitted for publication.
